# Supplementary material for: Cloud BioLinux: pre-configured and on-demand bioinformatics computing for the genomics community
Source: BMC Bioinformatics. 2012 Mar 19;13:42. doi: 10.1186/1471-2105-13-42 (PMC3372431; doi:10.1186/1471-2105-13-42)
Supplement: Additional file 1 — Supplementary 1 Cloud BioLinux software documentation in the form of a mini, self-contained website. Users need to download and uncompress the .zip file, and open through a web browser the "index.html" file available on the main directory. (ZIP 1823 kb). [file 1471-2105-13-42-S1.ZIP › Cloud-BioLinux-Package-Documentation/docs/run-mummer1.html]

Bio-Linux Software Documentation Pages

Back to search form

## run-mummer1

|  |  |
| --- | --- |
| Name | run-mummer1 |
| Description | **run-mummer1** is a part of the MUMmer package, for the rapid alignment of very large DNA and amino acid sequences.  **run-mummer1** is a legacy script from the original MUMmer1.0 release. It has been updated to utilize the new suffix tree code of version 3.0, however all other programs called from this script are identical to the original MUMmer release back in 1999. Even though it is an outdated program, it still has some advantages over the newer alignment scripts (nucmer, promer, run-mummer3).  Like all of the alignment scripts, run-mummer1 is a three step process - matching, clustering and extension. However, unlike the newer alignment scripts, run-mummer1 uses the gaps program for its clustering step. The gaps program does not allow for rearrangements like mgaps, instead if finds the single longest increasing subset of matches across the full length of both sequences. This makes it well suited for SNP and small indel identification between small (< 10 Mbp), very similar sequences with few to no rearrangements.  **References:**  Delcher AL, Kasif S, Fleischmann RD, Peterson J, White O, Salzberg SL: Alignment of whole genomes, Nucleic Acids Res. 1999 Jun 1;27(11):2369-76.[Entrez]    Delcher AL, Phillippy A, Carlton J, Salzberg SL: Fast algorithms for large-scale genome alignment and comparison, Nucleic Acids Res. 2002 Jun 1;30(11):2478-83.[Entrez]    Kurtz S, Phillippy A, Delcher AL, Smoot M, Shumway M, Antonescu C, Salzberg SL: Versatile and open software for comparing large genomes, Genome Biol. 2004;5(2):R12. Epub 2004 Jan 30.[Entrez] |
| Homepage | http://www.tigr.org/software/mummer/ |
| Remote Documentation | http://www.tigr.org/software/mummer/manual/ |
